# A Successful Conservative Management of Spontaneous Adrenal Hemorrhage (SAH) in Pregnancy: A Case Report

**DOI:** 10.7759/cureus.24989

**Published:** 2022-05-14

**Authors:** Waleed Almutairi, Ahmed Alibrahim, Mamdouh Alanazi

**Affiliations:** 1 Endocrinology and Metabolism, King Abdulaziz Medical City Riyadh, Riyadh, SAU

**Keywords:** non specific abdominal pain, spontaneous recovery, conservative management, pregnancy, spontaneous unilateral adrenal hemorrhage

## Abstract

It Is well known that abdominal pain during pregnancy has a broad differential diagnosis, which includes spontaneous adrenal hemorrhage (SAH), a rarely reported phenomenon in the literature, defined as an acute hemorrhage into the adrenal gland during pregnancy in the absence of a clear cause. Physicians should have a high suspicion for it due to the potentially life-threatening complications, as they present usually with a non-specific presentation. We present a case of symptomatic SAH in the third trimester of pregnancy that was successfully managed conservatively.

## Introduction

Spontaneous adrenal hemorrhage (SAH) is defined as hemorrhage occurring in the adrenal glands in the absence of preceding trauma, anticoagulant drug usage, or preexisting tumor [1]. A high index of suspicion is crucial for the diagnosis, as most patients present with nonspecific complaints such as abdominal or flank pain and fever [2]. Disastrous consequences for the mother and the baby can be prevented if detected and managed promptly.

## Case presentation

A 28-year-old female, with no known medical illness - gravida 4, para 3, abortus 0, pregnant at 36+4 weeks gestation - presented to the Emergency Department (ED) with a 1-day history of acute severe non-radiating abdominal pain that was sudden in onset, colicky in nature, and located at right lower quadrant area (she rated her pain as 9/10) - nothing seemed to aggravate or relive it - associated with nausea and two episodes of vomiting food content. She denied any history of fever, weight change, or trauma, also there were no urinary symptoms. A systematic review was otherwise unremarkable. Her previous pregnancies were uneventful and had no significant medical or surgical history.

On examination, she was an anxious and in-pain obese woman (BMI 34 kg/m²). She was mildly tachycardic (heart rate 108 beats per minute), but her other vital signs were within the normal range (Tables 1). Her abdomen circumference was consistent with gestational age, soft, and lax; the right flank tenderness along with guarding was appreciated. Otherwise, the rest of the examination was not contributory.

**Table 1:** Vital signs Abbreviations: SBP: systolic blood pressure, DBP: diastolic blood pressure, BP: blood pressure, HR: heart rate, RR: respiratory rate, T: temperature, SpO2: saturation of peripheral oxygen.

Item	Result
SBP (mmHg)	123
DBP (mmHg)	73
Mean BP (mmHg)	83
HR (Frequency/min)	108
RR (Frequency/min)	20
T (°C)	36.9
SpO2 (%)	100% on room air

Bedside pelvic/gynecological ultrasound was normal (i.e. normal gestational age, amniotic fluid index (AFI), and no clear signs of intraperitoneal hematoma). Cardiotocography (CTG) was reactive with no contractions. Apart from mild anemia (hemoglobin 96 gm/L - baseline 120 gm/L), leukocytosis (white blood cells 14.70 x10^9/L), and ketonuria, coagulation profile, and comprehensive metabolic panel were not significant (Tables 2-3).

**Table 2:** Laboratory results Abbreviations: Hgb: hemoglobin, WBC: white blood cells, Hct: hematocrit, MCV: mean corpuscular volume, MCH: mean corpuscular hemoglobin, RDW: red cell distribution width, INR: international normalized ratio, PT: prothrombin time, PTT: partial thromboplastin time, eGFR: estimated glomerular filtration rate, BUN: blood urea nitrogen, Adj Ca: adjusted calcium, CO2: carbon dioxide, Glu R: random glucose, Bili T: total bilirubin, Alk Phos: alkaline phosphatase, ALT: alanine aminotransferase, AST: aspartate aminotransferase.

Exam	Result	Reference Range
Hgb	96	120-160 gm/L
WBC	14.70	4-11 x10^9/L
Hct	0.312	0.36-0.54 L/L
MCV	79.7	76-96 fL
MCH	24.6	27-32 pg
RDW	16.4	11.5-14.5%
Platelet	188	150-400 x10^9/L
INR	0.95	0.80-0.120
PT	10.60	9.38-12.34
PTT	20.80	24.84-32.96
eGFR	166	>60 mL/min/1.73 m2
Creatinine	42	50-98 umol/L
BUN	1.6	2.5-6.7 mmol/L
Phosphorus	0.95	0.74-1.52 mmol/L
Adj Ca	2.18	2.1-2.55 mmol/L
Potassium	3.7	3.5-5.1 mmol/L
Sodium	136	136-145 mmol/L
CO2	19	22-29 mmol/L
Chloride	106	98-107 mmol/L
Glu R	5.8	2.9-7.8 mmol/L
Bili T	8.6	~ 20 umol/L
Alk Phos	159	40-150 u/L
Total Protein	65	64-83 g/L
Albumin	33	35-52 g/L
ALT	12	5-55 U/L
AST	24	5-34 U/L
Lipase Level	10	8-78 U/L
Amylase	73	25-125 U/L

**Table 3:** Urine analysis result Abbreviations: UA: urine analysis, Spec grav: specific gravity, pH: potential of hydrogen, Leuk Est: leukocytes esterase, WBC: white blood cell, Bili: bilirubin, RBC: red blood cells.

Exam	Result	Reference Range
UA Appear	Clear	Clear
UA Spec Grav	1.017	1.015-1.030
UA Color	Light-Yellow	Pale-Dark yellow
UA pH	5.5	5-7
UA Glucose	Negative	Negative
UA Ketones	>150	Negative
UA Blood	0.03	Negative
UA Leuk Est	Negative	Negative
UA Squam Epithelial	2	2-5 HPF
UA WBC	2	0-5 HPF
UA Bili	Negative	Negative
UA Urobilinogen	Trace	Trace
UA Nitrite	Negative	Negative
UA RBC	3	0-5 HPF
UA Mucous	+	None
UA Bacteria	Negative	Negative

**Figure 1:**
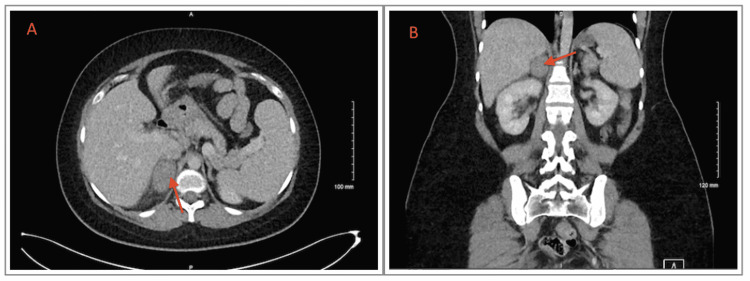
Axial (A) and coronal (B) views of CT scan demonstrate a right heterogeneous adrenal mass (arrows) consistent with hemorrhage.

In the Emergency Room (ER), after a detailed discussion regarding the risks and benefits, a contrast-enhanced low-dose abdomen and pelvis CT scan was done and showed no radiological evidence of appendicitis or nephrolithiasis, however, an abnormal adrenal finding was detected (Figure 1): the right heterogenous adrenal mass is seen measuring 2.7 x 4.5 cm (average Hounsfield units is 50~), no calcifications or macroscopic fat component, but had mild surrounding fatty stranding, no retroperitoneal hemorrhage. The left adrenal appeared unremarkable.

The patient was admitted under the Obstetrics and Gynecology team for observation and supportive measures, started on intravenous (IV) fluids (dextrose 5% in sodium chloride 0.9%) with analgesia (morphine and acetaminophen), in addition to antiemetics (metoclopramide) and antiacid (esomeprazole). The patient showed a good clinical response. 

The Endocrine Team was involved for the CT findings of adrenal hemorrhage. Upon assessment, no history or physical findings were suggestive of adrenal diseases, so the initial impression was that pheochromocytoma must be ruled out first. Serum and 24-hour urine metanephrines and normetanephrines came back negative. Morning cortisol was recommended but not drawn. Hence, the patient was discharged home with no steroid coverage and instructed to come back after 2 weeks for an elective cesarean section (due to breech position). Later, the surgery was done uneventfully and she delivered a healthy baby. 

During follow-up in the Endocrine clinic after 3 months, additional tests were sent (cortisol level after 1 mg dexamethasone suppression test and repeated metanephrines), which were noted all to be negative. An abdomen MRI was also arranged but not completed as our patient was claustrophobic.

**Figure 2:**
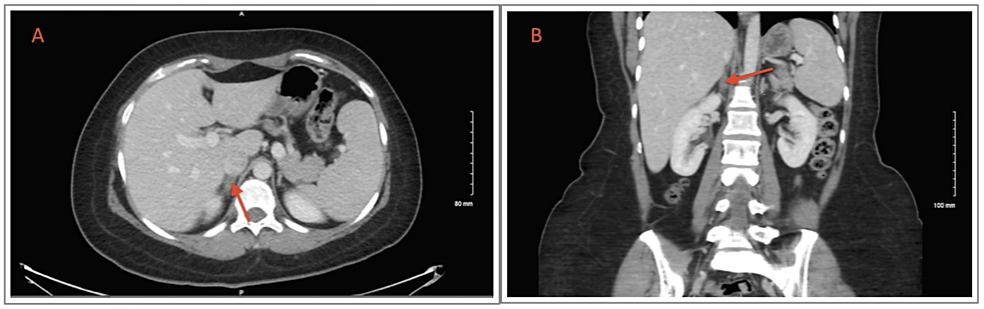
Axial (A) and coronal (B) views of CT scan demonstrate interval resolution of the previously seen right adrenal mass (arrows).

So, an abdomen and pelvis CT scan was performed instead and showed interval resolution of the previously seen right adrenal mass (Figure 2), which was likely representing pregnancy-related non-traumatic hemorrhage. The patient was counseled about her condition and reassured.

## Discussion

Spontaneous adrenal hemorrhage is considered to be a rare condition, as one autopsy report revealed that between 0.03 and 1.8% of unselected cases manifest adrenal hemorrhage. The incidence among pregnant women is unknown [3]. Fortunately, only a few patients develop massive retroperitoneal bleeding and present with hemodynamic instability [4]. A high index of suspicion is critical to prevent catastrophic maternal and fetal outcomes.

Presenting symptoms vary and are similar to those in non-pregnant patients and may include acute onset abdominal, flank, or even chest pain, nausea, vomiting, or low blood pressure [1]. In order to be classified as spontaneous, there should be no history of trauma, anticoagulation, tumor, or ongoing sepsis [2].

Suggested possible mechanisms for increased risk of SAH in pregnancy is adrenal cortex hyperplasia secondary to the physiological elevation of adrenocorticotropic hormone (ACTH) in addition to adrenal venous constriction owing to the increased catecholamine release [5]. 

The recommended laboratory evaluations in a suspected patient with SAH include serial hemoglobin measurements besides the assessment of adrenal function. Furthermore, as indicated, the workup should also be directed to rule out coagulopathy and thrombophilias, e.g. disseminated intravascular coagulation (DIC) and thrombotic thrombocytopenic purpura (TTP) [6]. Whilst the initial abdominal imaging study in pregnancy is preferred to be ultrasound, the sonographic features of adrenal hemorrhage are nonspecific. MRI is the most sensitive and specific imaging modality for confirming adrenal hemorrhage in pregnancy as well as evaluating for probable underlying etiology such as tumor or pheochromocytoma [2]. Nonetheless, CT scan can be an alternative method in specific situations like claustrophobia, non-compatible metallic orthopedic implants, and patients' preferences.

Management involves fetal monitoring, fluid resuscitation, pain control, close observation for findings suggestive of adrenal crisis, and glucocorticoid +/- mineralocorticoid replacement only as indicated. Moreover, blood transfusion and correction of coagulopathy, if present [5]. In case of hemodynamic instability with ongoing hemorrhage, arterial embolization can be considered, nevertheless, severely ill patients might warrant emergent adrenalectomy [7].

## Conclusions

Despite the fact that SAH is rare, it is a vital consideration when evaluating abdominal and flank pains in pregnancy. Poor outcomes and a high mortality rate for both mother and fetus can occur if left unrecognized, therefore, diagnosis requires a high index of suspicion, especially when the common etiologies of pain are excluded. Diagnosis can be established by MRI or CT scans. Based on observational studies, conservative management has favorable outcomes in a clinically stable patient, though more aggressive measures can be applied if necessitated.
